# Description of a new species of *Dacus* from Sri Lanka, and new country distribution records (Diptera, Tephritidae, Dacinae)

**DOI:** 10.3897/zookeys.795.29140

**Published:** 2018-11-08

**Authors:** Luc Leblanc, Camiel Doorenweerd, Michael San Jose, U.G.A.I. Sirisena, K.S. Hemachandra, Daniel Rubinoff

**Affiliations:** 1 University of Idaho, Department of Entomology, Plant Pathology and Nematology, 875 Perimeter Drive, MS2329, Moscow, Idaho, 83844-2329, USA University of Idaho Moscow United States of America; 2 University of Hawaii, Department of Plant and Environmental Protection Services, 3050 Maile Way, Honolulu, Hawaii, 96822-2231, USA University of Hawaii Honolulu United States of America; 3 Department of Plant Sciences, Faculty of Agriculture, Rajarata University of Sri Lanka, Puliyankulama, Sri Lanka Rajarata University of Sri Lanka Puliyankulama Sri Lanka; 4 Faculty of Agriculture, University of Peradeniya, Peradeniya, Sri Lanka University of Peradeniya Peradeniya Sri Lanka

**Keywords:** *
Bactrocera
*, Dacini, pest, taxonomy, *
Zeugodacus
*

## Abstract

A fruit fly survey in the Sinharaja and Knuckles National Parks in Sri Lanka (2016), using traps baited with the male lures methyl eugenol, cue-lure, and zingerone, yielded 21 species of Dacini fruit flies. Of these, three species, viz. *Bactroceraamarambalensis* Drew, *B.dongnaiae* Drew & Romig, and *B.rubigina* (Wang & Zhao), are new country occurrence records, and Dacus (Mellesis) ancoralis Leblanc & Doorenweerd, **sp. n.** is described as a new species. The Sri Lankan Dacini fruit fly fauna is now comprised of 39 species.

## Introduction

Dacine fruit flies are a large group of Old World tropical Diptera, with 932 described species, of which 83 are pests of fruit and fleshy vegetables (Vargas et al. 2015, Doorenweerd et al. 2018). Previous intensive surveys were carried out in Sri Lanka between 1993 and 1996 and resulted in a greatly improved understanding of the island’s diversity of fruit flies, including the description of eleven new species (Tsuruta et al. 1997, 2005, Tsuruta and White 2001). This increased the number of known species for Sri Lanka to 35 (Drew and Romig 2013, 2016). We report here the results of new surveys carried out twenty years after the last one, which include three new country occurrence records, and we describe one new species of *Dacus*.

## Materials and methods

We built traps from modified urine sample cups (described in Leblanc et al. 2015a) and deployed them along walking trails for 2–5 days in August 2015, to sample fruit flies in the Sinharaja National Park (40 sites) and the Knuckles National Park (31 sites). At each site, we maintained three traps, separately baited with the fruit fly lures methyl eugenol, cue-lure and zingerone. We used a 1×1 cm strip of dichlorvos insecticide to kill the flies entering traps. Collected specimens were preserved in 95% ethanol and later stored in a -20 °C freezer for long-term conservation, or double-mounted using 10 mm minuten pins. Prior to final mounting, we soaked minuten-pinned specimens for 3–12 hours in ethyl ether, to preserve the coloration. We identified flies using available keys (Tsuruta and White 2001, Drew and Romig 2016). We photographed the newly described species using a Nikon D7100 camera attached to an Olympus SZX10 microscope, with Helicon Focus pro v6.7.1 software used to stack photos taken at different focal planes. We took measurements using an ocular grid mounted on an Olympus SZ30 dissecting microscope. Morphological terms used in the description generally follow White (1999) and generic assignment for each species follows the checklist published by Doorenweerd et al. (2018). We extracted DNA and sequenced the mitochondrial COI-3P and COI-5P region from selected specimens to help confirm species identity. The entirety of the mitochondrial gene COI was sequenced using two PCR primers L1-DCHIM (5’-TCGCCTAAACTTCAGCCATT-3’) and PAT-K508 (5’-TCCAATGCAC and two additional internal primers (HCO-2198, 5’-TAAACTTCAGGGTGACCAAAAAATCA-3’ and HCO-2198RC 5’-TGATTTTTTGGTCACCCTGAAGTTTA-3’) resulting in a 1,535 base-pair (bp) long fragment. We sequenced up to six additional genes (two fragments of CAD, Wingless, White-eye, PGD, EF1-alpha and Period) for selected representatives of all species, including the one described here, and already available from a published dataset (San Jose et al. 2018). Pairwise genetic distance (p-distance) between specimens was calculated in Geneious R10.2.3. We performed maximum likelihood analysis of 1535 base-pairs of COI (3P + 5P region) of the newly described species and several closely related congeners using RaxML v8.2.11 (Stamatakis 2014). We repeated the best-scoring tree search 20 times and employed 1,000 multiparametric bootstrap searches with automatic halting following the extended majority rule criterion, halting the bootstraps after 200 searches, to estimate branch support. All specimen taxonomy and collecting data, as well as DNA sequences have been added to BOLD (dx.doi.org/10.5883/DS-DACANC) and the sequences have been deposited in Genbank (COI-5P: MH272136–MH272144 and COI-3P: MH272145–MH272155).

## Results

We collected a total of 3,498 specimens representing 21 species; 14 species in Sinharaja N.P. and 15 in Knuckles N.P. (Table 1). The majority (76.0 %) belong to seven pest species, dominated (64.5%) by *Bactrocerakandiensis* Drew and Hancock. We treat the previously described *Bactrocerainvadens* Drew, Tsuruta and White (Drew et al. 2005) as a junior synonym of *B.dorsalis* (see Schutze et al. 2015). Three species represent new country records: *B.rubigina* (Wang and Zhao), *B.dongnaiae* Drew and Romig, and *B.amarambalensis* Drew, and one new species, *Dacusancoralis* Leblanc & Doorenweerd, is described below, increasing the number of species known to occur in Sri Lanka to 39 (Table 1).

**Table 1.** Checklist of Dacine fruit flies of Sri Lanka, including three new country records and one new species, and number of specimens collected in Sinharaja and Knuckles National Parks in 2016.

Species	Distribution outside of Sri Lanka	PEST Status	Lure	Sinharaja N.P.	Knuckles N.P.
*B.amarambalensis* Drew*	Southern India	Non-pest	CL ^1^	0	1
*B.apicofuscans* White & Tsuruta	Southern India	Non-pest	ME	0	1
*B.bipustulata* (Bezzi)	Southern India	Non-pest	CL, ZN^2^	233	57
*B.brunneola* White & Tsuruta	Endemic to Sri Lanka	Non-pest	0	0	0
*B.ceylanica* Tsuruta & White	Endemic to Sri Lanka	Non-pest	CL	0	4
*B.correcta* (Bezzi)	Widespread in Asia	Fruit pest (polyphagous)	ME	10	58
*B.dongnaiae* Drew & Romig*	Vietnam	Non-pest	CL	3	0
*B.dorsalis* (Hendel)	Widespread in Asia, invasive in Africa and Oceania	Fruit pest (polyphagous)	ME	174	91
*B.fastigata* Tsuruta & White	Southern India	Non-pest	CL	0	0
*B.fernandoi* Tsuruta & White	Endemic to Sri Lanka	Non-pest	CL	0	0
*B.garciniae* Bezzi	Endemic to Sri Lanka	Non-pest	No lure	0	0
*B.hantanae* Tsuruta & White	Endemic to Sri Lanka	Non-pest	CL	12	0
*B.kandiensis* Drew & Hancock	Endemic to Sri Lanka	Fruit pest (polyphagous)	ME	1709	542
*B.latifrons* (Hendel)	Widespread in Asia, invasive in Africa and Oceania	Fruit pest (oligophagous)	Latilure	0	0
*B.nigrofemoralis* White & Tsuruta	Pakistan, India, Bhutan	Non-pest	CL	2	0
*B.nigrotibialis* (Perkins)	India to Malaysia	Fruit pest (oligophagous)	CL	0	0
*B.paraverbascifoliae* Drew	Southern India	Non-pest	ME	0	0
*B.perigrapha* White & Tsuruta	Bhutan	Non-pest	CL, ZN^2^	119	100
*B.profunda* Tsuruta & White	Endemic to Sri Lanka	Non-pest	CL	0	0
*B.rubigina* (Wang & Zhao)*	Bangladesh to Vietnam, Taiwan (new record)	Non-pest	CL, ZN^2^	118	0
*B.selenophora* Tsuruta & White	Endemic to Sri Lanka	Non-pest	CL	1	0
*B.syzygii* White & Tsuruta	Vietnam (new record)	Non-pest	ZN ^2^	42	144
*B.versicolor* (Bezzi)	Southern India	Fruit pest (sapodilla)	ME	0	0
*B.zonata* (Saunders)	Widespread in Asia, invasive in north Africa and Middle East	Fruit pest (polyphagous)	ME	0	1
*D.ancoralis* Leblanc & Doorenweld*	Endemic to Sri Lanka	Non-pest	ZN ^2^	1	0
*D.ciliatus* Loew	Africa, Middle East, Indian subcontinent	Cucurbit fruit pest	No lure	0	0
*D.discophorus* (Hering)	Southern India	Non-pest	CL, ZN^2^	0	1
*D.keiseri* (Hering)	Endemic to Sri Lanka	Non-pest	No lure	0	0
*D.persicus* Hendel	Middle East, Pakistan, India	Non-pest	No lure	0	0
*D.ramanii* Drew & Hancock	Southern India	Non-pest	CL	0	0
*Z.caudatus* (Fabricius)	Widespread in Asia	Cucurbit flower pest	CL	0	1
*Z.cucurbitae* (Coquillett)	Widespread in Asia, invasive in Africa and Oceania	Cucurbit fruit pest	CL	50	9
*Z.diaphorus* (Hendel)	Widespread in Asia	Non-pest	CL	0	0
*Z.diversus* (Coquillett)	Pakistan to Thailand	Cucurbit flower pest	ME	0	0
*Z.duplicatus* (Bezzi)	Southern India	Non-pest	No lure	0	0
*Z.gavisus* (Munro)	India	Non-pest	CL	0	0
*Z.tau* (Walker)	Widespread in Asia	Cucurbit fruit pest	CL	12	1
*Z.trilineatus* (Hardy)	India, Thailand, Vietnam	Non-pest	CL	0	1
*Z.zahadi* (Mahmood)	Pakistan to Myanmar	Non-pest	CL	0	0

* New country occurrence records. ^1^ Uncertain lure record (see text). ^2^ New lure records. Lure abbreviations: CL = cue-lure, ME = methyl eugenol, ZN = zingerone

One species not attracted to the traditional male lures, *B.syzygii* White & Tsuruta, was captured in zingerone-baited traps, as well as cue-lure responding *B.bipustulata* (Bezzi), *B.perigrapha* White & Tsuruta, *B.rubigina* (Wang & Zhao), and *Dacusdiscophorus* (Hering). The new species was also collected at zingerone. The specimen identified as *B.amarambalensis* Drew was collected in a cue-lure trap, whereas the two other known specimens (holotype and paratype) were collected at methyl eugenol. This may represent lure contamination or a superficially very similar species that shares the markings on postpronotal lobes and other distinctive features of the described species.

In addition to fruit flies, lacewings (Neuroptera: Chrysopidae) were collected in the methyl eugenol traps (133 specimens in Sinharaja N.P. and 55 in Knuckles N.P.). All specimens are consistent with *Ankylopteryxanomala*, a widely distributed species previously recorded from Sri Lanka (Breitkreuz et al. 2015), and for which attraction to methyl eugenol is well documented (Leblanc et al. 2015b).

### Dacus (Mellesis) ancoralis

Taxon classificationAnimaliaDipteraLauxaniidae

Leblanc & Doorenweerd sp. n.

http://zoobank.org/1AA99C05-4095-45F3-B508-774FAC0414C4

#### Holotype.

Male. Labeled: “Sri Lanka: Sinharaja Forest Reserve, 6.3645N, 80.4786E, 22–24-viii-2016, D. Rubinoff, M. San Jose and U.G.A.I. Sirisena, FF638, zingerone trap, molecular voucher ms7321.” Deposited in the University of Hawaii Insect Museum (UHIM).

#### Differential diagnosis.

*Dacusancoralis* is similar to other Asian species of *Dacus* with a red-brown scutum lacking the yellow medial and lateral vittae and with a costal band of uniform width that crosses vein R_4+5_ over the entire length of the wing, but does not reach vein M, and fuscous cells bc and c, such as *Dacuspolistiformis* (Senior-White), *D.wallacei* White, *D.longicornis* Wiedemann, *D.insulosus* Drew and Hancock and *D.discretus* Drew and Romig. *Dacusancoralis* differs from *D.polistiformis* and *D.wallacei* in lacking spines on the femur of the front legs, and it differs from all other aforementioned species by having dark fulvous postpronotal lobes. The closely related *D.vijaysegarani* (Figure 2A–E) Drew and Hancock is easily separated by its black scutum, mostly black abdomen and black legs.

**Figure 1.**
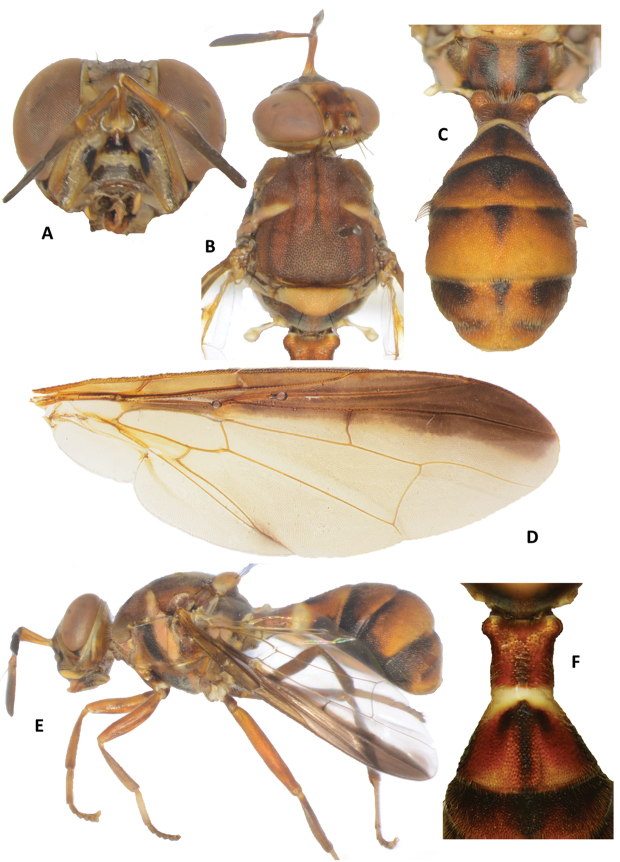
Dacus (Mellesis) ancoralis sp. n. **A** head **B** head and scutum **C** abdomen **D** wing **E** lateral view **F** Abdominal tergum II, with anchor-shaped marking.

**Figure 2.**
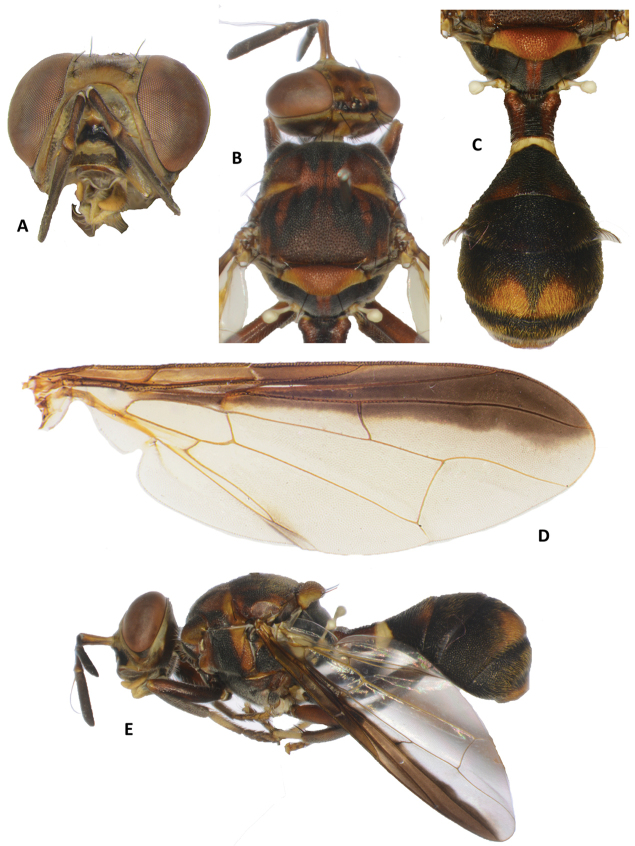
Dacus (Mellesis) vijaysegerani (Drew and Hancock). **A** head **B** head and scutum **C** abdomen **D** wing **E** lateral view.

#### Molecular diagnostics.

**Figure 3.**
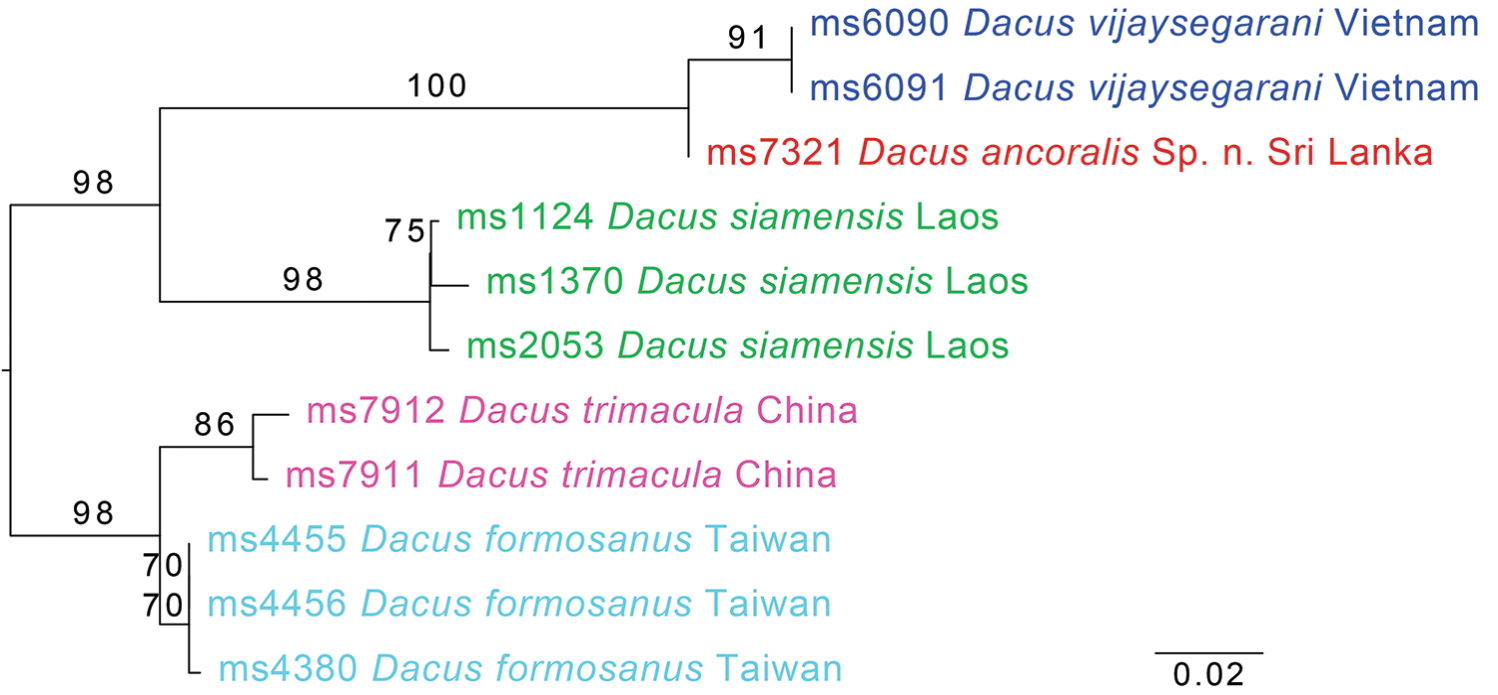
Maximum likelihood tree based on COI (1535 base-pairs) sequence data. Bootstrap support is indicated on the respective branches. Scale bar indicates substitutions per site.

Figure 3 shows the maximum likelihood tree based on combined COI-5P and COI-3P regions (1535 base-pairs [bp]) for *Dacusancoralis* and the closest congeners in our dataset. In the COI-3P fragment (836 bp), the minimum p-distance to *Dacusvijaysegarani* is 1.38%, and in the COI-5p DNA barcode fragment (658 bp) it is 2.43%. Because there is only one specimen of *D.ancoralis* we cannot test for reciprocal monophyly. The overall next closest relative in our dataset is *D.siamensis*, at a minimum p-distance of 8.61% in COI-3P, 8.81% in COI-5P.

#### Description of adult.

*Head* (Figure 1A). Vertical length 2.00 mm. Frons, of even width, length 1.06 times as long as broad; red-brown with fuscous around orbital setae and on anteromedial hump; latter covered by short red-brown hairs; orbital setae dark fuscous: one pair of superior and two pairs of inferior fronto-orbital setae present; lunule fulvous. Ocellar triangle dark fuscous. Vertex fuscous. Face fulvous with medium sized oval black spots in each antennal furrow, a fuscous band along lower margin between spots and a dark fuscous triangular marking below antennal sockets; length 0.55 mm. Genae red-brown, with fuscous subocular spot; dark fuscous seta present. Occiput fulvous and yellow along eye margins; occipital row with two parallel rows of adjacent setae (with 11 and 17 setae). Antennae with segments 1 (scape) and 2 (pedicel) fulvous and segment 3 (first flagellomere) fuscous; a strong red-brown dorsal seta on segment 2; arista black (fulvous basally); length of segments: 0.83 mm; 0.70 mm; 1.23 mm.

*Thorax* (Figure 1B, E). Scutum red-brown with a broad light fuscous lanceolate pattern on its posterior third, anteriorly prolonged into three very narrow lines reaching anterior margin, light fuscous narrow outer bands parallel to lanceolate pattern and reaching notopleural suture. Pleural areas red-brown except a broad vertical dark fuscous band in front of mesopleural stripe, a large dark fuscous spot occupying central portion of katepisternum, and a dark fuscous spot on katepimeron above hind coxa. Yellow markings as follows: notopleura (notopleural callus); narrow parallel-sided mesopleural (anepisternal) stripe, reaching midway between anterior margin of notopleura and anterior notopleural seta dorsally, continuing to katepisternum as a transverse spot and to scutum as moderately broad yellow markings along anterior margin of notopleural suture; lower 25% of anatergite (remainder dark fulvous); anterior 70 % of katatergite (remainder black). Postpronotal lobes dark fulvous. Medial and lateral postsutural vittae absent. Postnotum red-brown with two broad longitudinal fuscous bands. Scutellum yellow except for narrow black basal band. Setae (number of pairs): 1 scutellar; prescutellar absent; 1 intraalar; 1 posterior supraalar; 1 anterior supraalar; 1 mesopleural; 2 notopleural; 4 scapular; all setae well developed and black.

*Legs* (Figure 1E). Femora and tibiae orange-brown, except for fuscous ventral surface of hind femur; mid-tibiae each with an apical black spur; tarsi fulvous.

*Wings* (Figure 1D). Length 7.00 mm; basal costal (bc) and costal (c) cells fuscous and covered with microtrichia; remainder of wings colorless except dark fuscous subcostal cell, broad dark fuscous costal band overlapping R_4+5_ and of uniform width, not reaching vein M; anal streak absent; supernumerary lobe weakly developed.

*Abdomen* (Figure 1C, E, F). Elongate, clavate and petiolate; terga tightly joined but with medial protuberances; pecten of cilia present on tergum III; posterior lobe of surstylus short; abdominal sternum V with a slight concavity on posterior margin. Tergum I and sterna I and II longer than wide. Tergum I orange-brown with apical third yellow and a median light fuscous band on apical half of red-brown portion. Tergum II orange-brown with medial dark fuscous narrow band and two short basal bands, lateral to medial band, forming an anchor-shaped pattern, and broad fuscous markings on lateral margins. Tergum III orange-brown with dark fuscous as along base and extended to whole lateral margins and into a triangular medial band. Tergum IV orange-brown with dark fuscous medial basal triangular marking, narrowly along base of tergum and broadly along entire lateral margins. Tergum V orange-brown with dark fuscous medial basal triangular marking, and large lateral bands covering basal half of tergum and reaching lateral margins. A pair of basally fuscous and apically orange-brown ceromata (shining spots) on tergum V. Abdominal sterna dark except pale sternite II.

#### Etymology.

The name *ancoralis* is a noun in apposition that refers to the anchor-shaped fuscous pattern on abdominal tergum II in the holotype (Figure 1F).

#### Notes.

Although *Dacusancoralis* is genetically closely related to *D.vijaysegarani* and there is only one specimen, they do not appear to be sympatric, with *D.vijaysegarani* only known from Malaysia, Thailand and Vietnam, and with the clear differences in coloration of all body parts we are confident in describing it as a new species. The holotype of *Dacusancoralis* was referred to as “ms7321 Dacus (Mellesis) sp-78”, sister to *D.vijaysegarani*, in the seven-gene phylogeny presented in San Jose et al. (2018). It keys to couplet 37 (p 467) in the Keys to the Fruit Flies of South-East Asia (Drew and Romig 2016), where it can be added as a unique combination of having dark fuscous postpronotal lobes and a red-brown scutum. *Dacusancoralis* was collected in a trap with zingerone lure. A number of other species of *Dacus* were found to be drawn to zingerone in recent years (Doorenweerd et al. 2018), but because there is only one specimen known we cannot yet confirm it as a zingerone-attracted species. This species is assigned to subgenus Mellesis, as defined by Drew and Romig (2013) based on the petiolate abdomen with tergum I longer than wide and sternum V weakly concave apically, the presence of anterior supraalar setae and absence of prescutellar setae, the combined length of antennal segment greater than vertical length of face, and the absence of anal streak on wing. Its nearest relatives all belong to subgenusMellesis (Figure 3).

## Supplementary Material

XML Treatment for Dacus (Mellesis) ancoralis
